# Efficacy of SGLT2 inhibitors as additional treatment in Japanese type 2 diabetic patients: second or third choice?

**DOI:** 10.1186/s13104-022-06010-6

**Published:** 2022-03-29

**Authors:** Makoto Fujiwara, Masaru Shimizu, Yuko Maejima, Kenju Shimomura

**Affiliations:** 1 Department of Diabetes, Endocrinology and Metabolism, Tsukuba Medical Center, Ibaragi, Japan; 2 Department of Bioregulation and Pharmacological Medicine, Fukushima Medical University School of Medicine, 1 Hikarigaoka, Fukushima, 960-1295 Japan; 3 Department of Neurology, Matsumura General Hospital, Fukushima, Japan

**Keywords:** SGLT2 inhibitors, HbA1c, Metformin, DPP4 inhibitor

## Abstract

**Objectives:**

Due to the increase of type 2 diabetes (T2D), the number of patients in treatment with multiple anti-diabetic agents is increased. According to the recent recommendation of treatment guidelines, sodium-glucose cotransporter 2 (SGLT2) inhibitors would be used as additional treatment to the currently administered anti-diabetic drugs for poorly controlled T2D patients. Here, we assessed the efficacy of SGLT2 inhibitors added to the current treatment with metformin, dipeptidyl peptidase-4 (DPP4) inhibitors, or both in Japanese T2D patients.

**Results:**

Japanese T2D subjects with poor glucose control, who were treated with metformin (n = 10), DPP4 inhibitors (n = 11), or both (n = 28) and who were in need of additional treatment, were recruited. HbA1c levels before and 6 months after addition of SGLT2 inhibitor treatment were used to compare the effectiveness. The HbA1c levels after addition of SGLT2 inhibitors significantly decreased in all groups. The change in HbA1c levels (delta HbA1c) showed no significant difference between the three groups. The present data indicated that addition of SGLT2 inhibitors to metformin and/or DPP4 inhibitors is equally effective in the treatment of Japanese T2D patients.

## Introduction

When diet and exercise treatments are not sufficient to obtain good glycemic control in type 2 diabetes (T2D) patients, treatment guidelines recommend initiation of treatment with single oral hypoglycemic agent [1, 2]. A Japanese guideline recommends that the initial anti-diabetic agent should be selected based on the individual condition of patients [1]. The majority of East Asian T2D patients, including Japanese, present insulin secretion defect rather than insulin resistance [3]. Insulin secretion capacity in Japanese T2D patients has shown to be half compared to that in Caucasian patients [4]. Therefore, dipeptidyl peptidase-4 (DPP4) inhibitors, which promote insulin secretion and have a well characterized safety profile, are widely used in Japan. In addition, the American Diabetes Association (ADA) guidelines recommend metformin, which improves insulin resistance, as the initial oral anti-diabetic agent, and metformin is also widely used in Japan [2].

However, single anti-diabetic oral drug gradually becomes less effective in obtaining good glycemic control, and additional treatment is required. Currently, many Japanese T2D patients undergo combination treatment with DPP4 inhibitors and metformin, which are most commonly used anti-diabetic drugs. Although this combination treatment is effective, its efficacy decreases over time, and a third drug is required to obtain good glycemic control.

Sodium-glucose cotransporter-2 (SGLT2) inhibitors are a new class of anti-diabetic drugs and have glucose lowering effects by inhibiting the renal reabsorption of glucose and increasing glucosuria [5, 6].

Considering the pharmacological action of SGLT2 inhibitors, which differs from that of DPP4 inhibitors and metformin, the additional improvement of glycemic control is expected when SGLT2 inhibitors are added to DPP4 inhibitors or metformin. However, it remains unclear whether the effectiveness of SGLT2 inhibitors differs when added to monotherapy or combination therapy of DPP4 inhibitors and metformin in T2D patients.

Here, we assessed the efficacy of SGLT2 inhibitors added to the current treatment with DPP4 inhibitors, metformin, or both in Japanese T2D patients.

## Main text

### Methods

#### Patient information

This is a retrospective cohort study including outpatients of Tsukuba Medical Center and Matsumura General Hospital for diabetes.

Thirty-four subjects (18 men and 16 women) treated with combination therapy of DPP4 inhibitors and metformin, 11 subjects (6 men and 5 women) treated with DPP4 inhibitors and 10 subjects (4 men and 6 women) treated with metformin were recruited. The length of diabetes in DPP4 inhibitor and metformin combination treated group was 23 subjects for more than 10 years, 5 subjects for 5 years, 3 subjects for 2 years and 3 subjects for a year. The length of diabetes in only DPP4 inhibitor treated group was 3 subjects for more than 10 years, 1 subject for 8 years, 3 subjects for 5 years, 1 subject for 4 years and 3 subjects for 2 years. The length of diabetes in only metformin treated group was 6 subjects for more than 10 years, 1 subject for 9 years, 1 subject for 9 years, 1 subject for 3 years and 2 subjects for 2 years. Some of the subjects recruited in this study had other metabolic disorders (hyperlipidemia and/or hyperuricemia). Three subjects had hyperlipidemia, 1 subject had hyperuricemia and 2 subjects had both for DPP4 inhibitor treatment group. Nine subjects had hyperlipidemia, 0 subject had hyperuricemia and 1 subject had both for the metformin treatment group. Seventeen subjects had hyperlipidemia, 0 subject had hyperuricemia and 4 subjects had both for combination treatment group.

All the patients recruited in this study who are using metformin were treated with 500 mg/day. The DPP4 inhibitors used by the subjects in this study were alogliptin, sitagliptin, linagliptin, vildagliptin, anagliptin, saxagliptin, tenegliptin. All DPP4 inhibitors were used in standard dosage according to the drug protocol. Drugs are all administered orally.

#### Research methods

HbA1c levels were used to assess the effectiveness of adding SGLT2 inhibitors to the current treatment. HbA1c levels were measured before and 6 months after adding SGLT2 inhibitors. SGLT2 inhibitors were added in the patients who had HbA1c levels higher than 6.9 in spite of the DPP4 inhibitors and metformin treatment. Added SGLT2 inhibitors were ipragliflozin, canagliflozin, dapagliflozin, luseogliflozin or empagliflozin. All SGLT2 inhibitors were used in standard dosage according to the drug protocol.

#### Statistical analysis

All data are presented as mean ± SEM. Student’s *t*-test was used for two-group comparisons. All statistical tests were two-tailed, with values of 0.05 being considered statistically significant.

## Results

The average age of the 11 patients treated with DPP4 inhibitors, the 10 patients treated with metformin, and the 34 patients treated with the combination of those two drugs was 56.3 ± 7.4, 53.0 ± 3.2, and 55.1 ± 1.7 years old, respectively, showing no significant difference.

The average body mass index of each group were not significantly different: 26.0 ± 2.3 in the DPP4 inhibitor treatment group, 26.8 ± 1.2 in the metformin treatment group, and 26.1 ± 1.2 in the combination treatment group.

HbA1c levels before the addition of SGLT2 inhibitors were approximately the same among the three groups; 8.7 ± 0.7% in the DPP4 inhibitor treatment group, 8.6 ± 0.5% in the metformin treatment group, and 8.5 ± 0.3% in the combination treatment group.

**Fig. 1:**
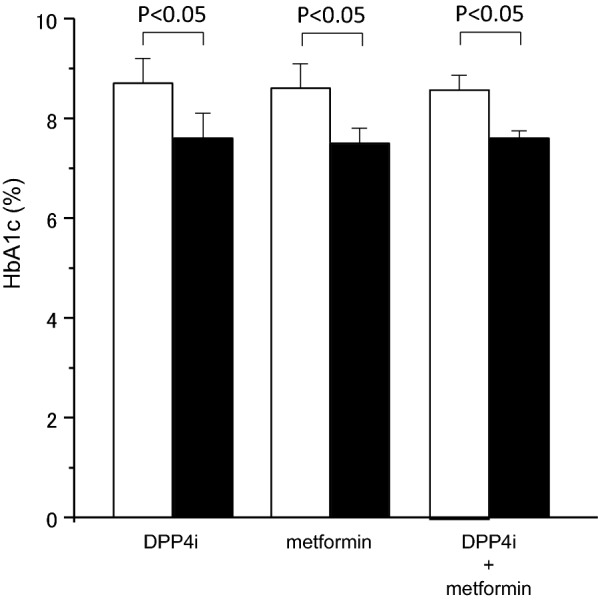
HbA1c levels before and after the addition of SGLT2 inhibitors. Open boxes indicate before the SGLT2 treatment and closed boxes indicate after the SGLT2 treatment

Addition of SGLT2 inhibitors significantly improved HbA1c levels to 7.6 ± 0.5%, 7.5 ± 0.3% and 7.6 ± 0.2%, respectively (Fig. 1).

**Fig. 2:**
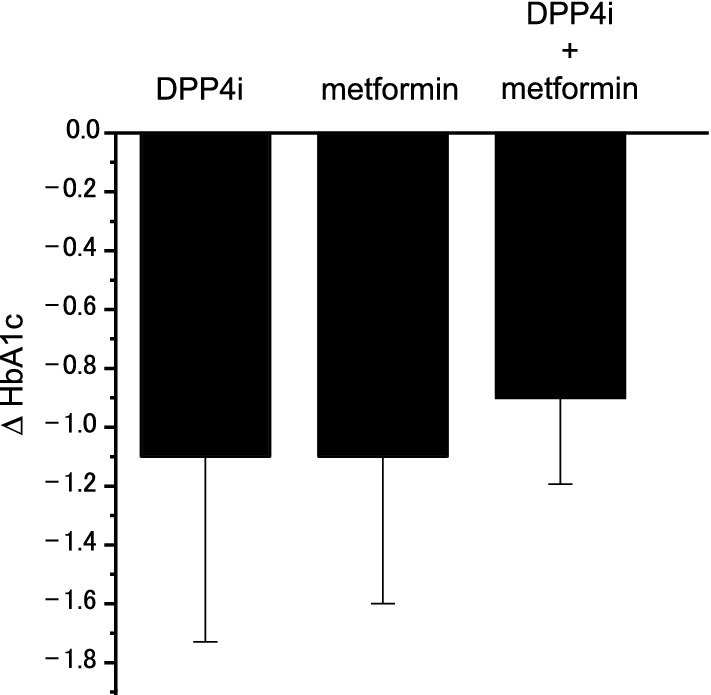
Changes in HbA1c levels (Δ HbA1c) after the addition of SGLT2 inhibitors

When comparing the reduction of HbA1c levels before and after the additional application of SGLT2 inhibitors, no significant difference was observed in three groups, which was − 1.1 ± 0.6 for the DPP4 inhibitor group, − 1.1 ± 0.5 for the metformin group and − 0.9 ± 0.3 for the combination group (Fig. 2). The data indicate that the effect of SGLT2 inhibitors added to DPP4 inhibitor/metformin monotherapy or their combination is similar in Japanese T2D patients.

## Discussion

The increase in the number of T2D patients is a global problem, and Japan is not an exception. However, a characteristic for the development of hyperglycemia is reported to be different between patients in Europe/US and East Asia [3, 7–9]. The report from Diabetes epidemiology: collaborative analysis of diagnostic criteria I Europe (DECODE) and in Asia (DECODA) studies revealed a predominance of insulin resistance with obesity in T2D patients in Europe and US, whereas impaired insulin secretion is predominant in East Asia [10–12].

The mechanism for impaired insulin secretion observed in T2D patients from East Asia can be explained by the result from clinical study in Japan showing that intact level of glucagon-like peptide-1 (GLP-1), a hormone which stimulate insulin secretion under hyperglycemic condition and contribute to nearly 70% of the post-prandial insulin secretion, being considerably low in both healthy and T2D subjects compared to Caucasian [13–15]. Therefore, DPP4 inhibitors that prevent the degradation of GLP-1 are considered as an effective agent to treat Japanese T2D patients. In fact, it is reported in the past that DPP4 inhibitors are prescribed in around 50% of diabetic patients in Japan as a first choice and prescribed in approximately 75–80% of previously treated Japanese diabetic patients [16].

On the other hand, metformin, which improves insulin resistance, is also reported to have a positive effect on Japanese T2D patients and widely used for its high efficacy, low cost and low risk of side effects [17].

A considerable number of Japanese T2D patients are therefore under treatment with DPP4 inhibitors, metformin, or both. The HbA1c lowering effect of DPP4 inhibitors and that of metformin are reported to be similar in Japanese T2D patients [18, 19].

The recent addition of SGLT2 inhibitors widened the selection of anti-diabetic drugs to treat T2D patients. Clinical trials such as EMPA-REG OUTCOME even raised the possibility of positive effects of SGLT2 inhibitors on cardiovascular outcome [20].

Based on the study analyzing the data from Japan Medical Data Centre, it is reported that the probability on remaining on treatment with combination of two different antidiabetic drug for 12 month remain to 40–54% [16, 21]. Therefore it can be considered that the need of adding third drug to gain proper glycemic control is rather common in Japan.

In this study we assessed the efficacy of SGLT2 inhibitors added to currently administered DPP4 inhibitors, metformin, or both in T2D patients. Our present data showed that additional SGLT2 inhibitors had a similar effect on lowering HbA1c levels in T2D patients treated with DPP4 inhibitor, metformin, or both.

The addition of SGLT2 inhibitor on patient treated with combination of DPP4 inhibitor and metformin showed similar effect compared to adding SGLT2 inhibitor on monotherapy group. One possibility is that DPP4 inhibitor and metformin combination group consisted with patients with longer duration of diabetes period (23 subjects were diabetes for more than 10 years). Further study on influence of the diabetes duration is required in the future.

The present study indicates that the addition of SGLT2 inhibitors is effective in Japanese T2D patients who are unable to obtain good glycemic control with DPP4 inhibitors or metformin, or both. Therefore, the addition of SGLT2 inhibitors should not be hesitated for glycemic control improvement in such patients.

## Limitations

Due to a small sample size of subjects, further study is required. Also, due to the small sample size we were unable to compare the effect of adding SGLT2 inhibitors on different DPP4 inhibitors. Further study is also required.

## Data Availability

The datasets used and/or analyzed during the current study are available from the corresponding author on reasonable request.

## Abbreviations

SGLT2: Sodium-glucose cotransporter 2
DPP4: Dipeptidyl peptidase-4
T2D: Type 2 diabetes
